# Supplementary material for: Lysine acetylation regulates the interaction between proteins and membranes
Source: Nat Commun. 2021 Nov 9;12:6466. doi: 10.1038/s41467-021-26657-2 (PMC8578602; doi:10.1038/s41467-021-26657-2)
Supplement: Supplementary file 1 — Supplementary Information [file 41467_2021_26657_MOESM1_ESM.pdf]

Lysine Acetylation Regulates the Interaction between Proteins and Membranes

Alan K. Okada[1*], Kazuki Teranishi[2*], Mark R. Ambroso[2*], Jose Mario Isas[2*], Elena Vazquez Sarandeses[3,], Joo-Yeun Lee[4], Arthur Alves Melo[3], Priyatama Pandey[5], Daniel Merken[5], Leona Berndt[6], Michael Lammers[6], Oliver Daumke[3], Karen Chang[2,4], Ian S. Haworth[5], and Ralf Langen[2,¥]

[1]Regions Hospital Department of Emergency Medicine, Saint Paul, Minnesota 55101, [2]Zilkha Neurogenetic Institute, Department of Physiology and Neuroscience, University of Southern California, Los Angeles, California 90033, USA, [3]Max-Delbrück-Center for Molecular Medicine, Crystallography, Robert-Rössle-Straße 10, 13092 Berlin, Germany; Institute of Chemistry and Biochemistry, Freie Universität Berlin, Takustraße 6, 14195 Berlin, Germany; Institute of Medical Physics and Biophysics, Charité, Charitéplatz 1, 10117 Berlin, Germany, [4]Neuroscience Graduate Program, University of Southern California, Los Angeles, California, USA, [5]Department of Pharmacology and Pharmaceutical Sciences, University of Southern California, Los Angeles, California 90089, USA, [6]Institute of Biochemistry, Synthetic and Structural Biochemistry, University of Greifswald, 17487 Greifswald, Germany.

[¥]Corresponding Author: Ralf Langen, Tel.: 323-442-1323 Fax: 323-442-4404. Email: Langen@usc.edu

*These authors contributed equally to this research.

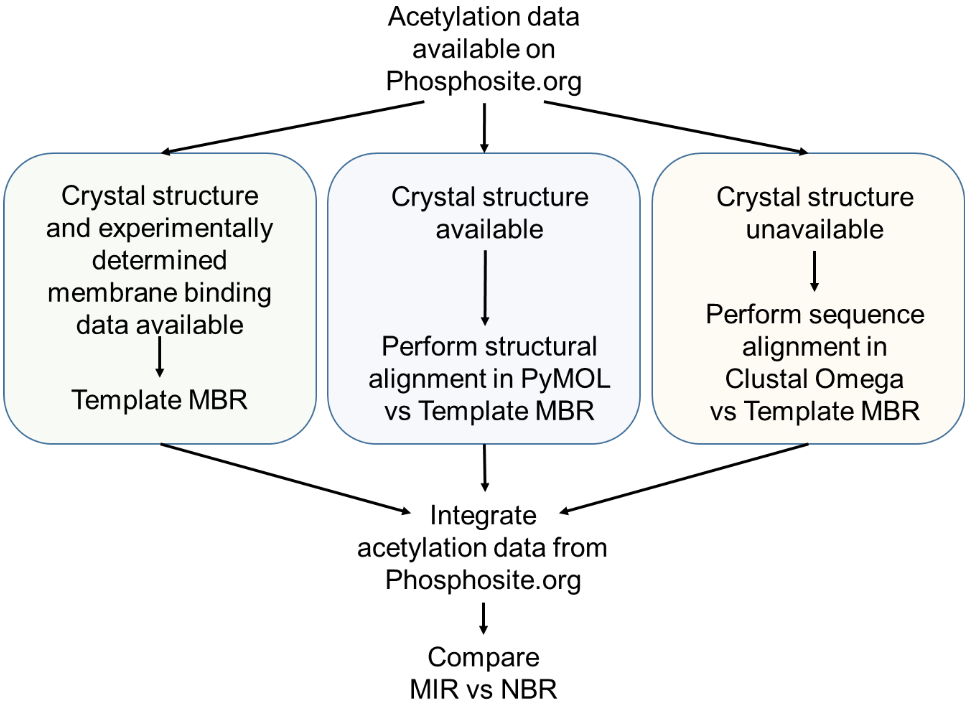

Supplementary Figure 1. **Schematic of the approach used to determine the differential distribution of acetylation within membrane-binding domains.** MIR, membrane-interaction region; NBR, non-binding region.

a

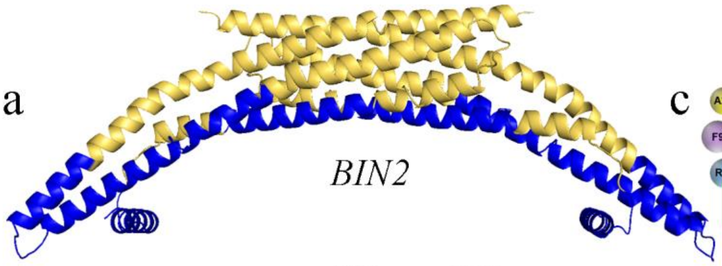

b

Gene name (.pdb)	MIR $\%K_{AC}$	NBR $\%K_{AC}$	Ratio
AMPH (1URU)	6.25	1.39	4.50
SH3GL2 (2C08)	1.98	0.70	2.83
SH3GL3 (2Z0V)	0.99	0.00	MIR only
BIN1 (2FIC)	1.53	0.72	2.11
BIN2 (4AVM)	7.27	0.00	MIR only
SNX9 (3DYT)	1.45	0.00	MIR only
SNX33 (4AKV)	2.86	0.00	MIR only
FNBP1 (2EFL)	4.96	0.76	6.55
FCHO2 (2V0O)	1.45	1.65	0.88
TRIP10 (2EFK)	1.40	0.00	MIR only
PACSIN1 (2HAI)	6.49	1.45	4.48
PACSIN2 (2HAJ)	1.30	0.73	1.78
Average	3.20	0.61	5.21

c

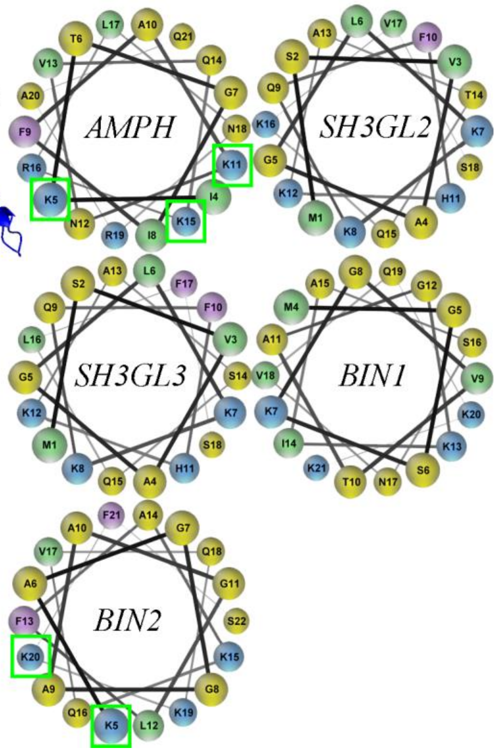

Supplementary Figure 2. **Acetylation within BAR domains is localized to membrane-interaction regions.** A) *BIN2* (4AVM.pdb) structure displayed to illustrate MIRs and NBRs colored blue and gold, respectively. B) BAR domain acetylation prevalence listed by associated BAR domain containing gene. Percentages represent the number of acetylated lysine residues normalized by the total number of amino acid residues in their respective regions. (.pdb) file names indicate the PDB crystal structure used in the analysis. See Supplementary spreadsheet 1 for details including definitions of domains, MIRs and NBRs. C) H0 N-terminal helices of N-BAR domains are represented as 18 amino acid helical wheels. Acetylated residues are highlighted with a green box. Source data are provided as a Source Data file.

a

b

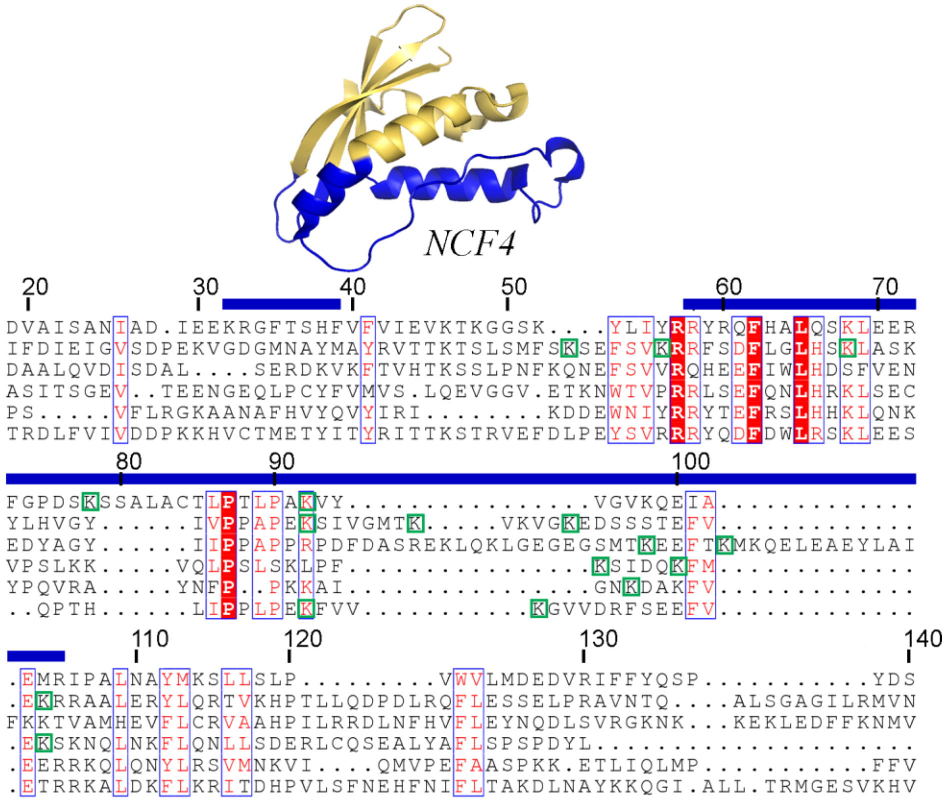

c

Gene name (.pdb)	MIR $\%K_{AC}$	NBR $\%K_{AC}$	Ratio
NCF4 (1H6H)	3.57	0.00	MIR only
SNX1 (2I4K)	1.59	0.00	MIR only
SNX5 (3HPC)	0.00	1.52	0.00
SNX9 (3DYT)	2.33	0.00	MIR only
SNX12 (2CSK)	0.00	1.47	0.00
SNX14 (4PQO)	5.45	0.00	MIR only
SNX19 (4P2I)	0.00	1.35	0.00
SNX2	8.20	2.30	3.57
SNX6	2.56	0.00	MIR only
SNX25	5.88	0.00	MIR only
SNX29	2.04	0.00	MIR only
SNX30	3.77	0.00	MIR only
Average	2.82	0.57	4.92

Supplementary Figure 3. **Acetylation within PX domains is localized to membrane-interaction regions.** A) p40^{phox} structure (1H6H.pdb) with MIRs and NBRs colored blue and gold, respectively. B) Sequences of PX domains aligned against p40phox (residues 19-140). The blue bar denotes the MIR. Acetylation sites are marked by green boxes. C) Acetylation prevalence among PX domain containing genes. Percentages represent the number of acetylated lysine residues out of the total amino acid residues in their respective regions. (.pdb) file names indicate the PDB crystal structure used. See Supplementary spreadsheet 1 for details including definitions of domains, MIRs and NBRs. Source data are provided as a Source Data file.

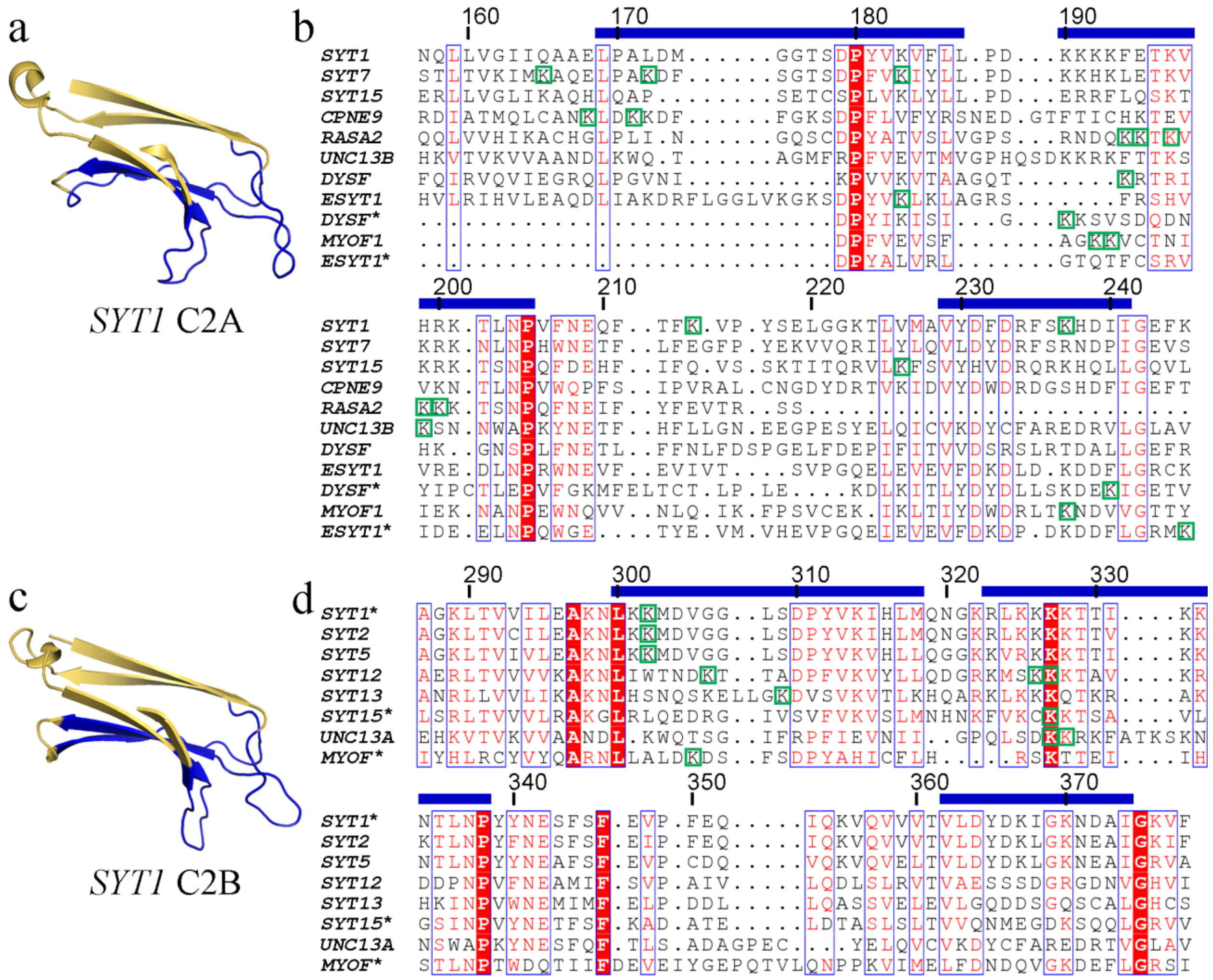

e

Gene name (.pdb)	MIR %K_{AC}	NBR %K_{AC}	Ratio
SYT1 (2R83)	2.08	2.44	0.85
PRKCA (4DNL)	4.17	2.38	1.75
PIK3C2A (2B3R)	2.13	0.00	MIR only
SYT7	4.17	2.38	1.75
SYT15	0.00	2.44	0.00
CPNE9	2.08	2.22	0.94
RASA2	14.71	0.00	MIR only
UNC13B	2.13	0.00	MIR only
DYSF	2.56	0.00	MIR only
ESYT1	2.04	0.00	MIR only
*DYSF**	5.26	0.00	MIR only
MYOF1	8.11	0.00	MIR only
*ESYT1**	0.00	4.00	0.00
Average	3.54	1.22	2.90

f

Gene name (.pdb)	MIR %K_{AC}	NBR %K_{AC}	Ratio
*SYT1**(2R83)	2.08	0.00	MIR only
PRKCG (2UZP)	4.00	0.00	MIR only
SYT2	2.08	0.00	MIR only
SYT5	2.08	0.00	MIR only
SYT12	6.25	0.00	MIR only
SYT13	2.00	0.00	MIR only
*SYT15**	2.13	0.00	MIR only
UNC13A	3.92	0.00	MIR only
*MYOF**	2.27	0.00	MIR only
Average	3.00	0.00	MIR only

Supplementary Figure 4. **Acetylation within C2 domains is localized to membrane-interaction regions.** A & C) Synaptotagmin1 C2A and C2B domain structures (2R83.pdb) with MIRs and NBRs colored blue and gold, respectively. B & D) Sequence alignments of C2A (157-245) and C2B (287-378) domains are shown with blue bars denoting MIRs and green boxes around acetylation sites. E & F) Acetylation prevalence in MIRs and NBRs are shown by gene for domains aligned with C2A (e) or C2B (f). Percentages represent the number of acetylated lysine residues out of the total amino acid residues in their respective regions. (.pdb) file names indicate the PDB crystal structure used. * indicates a different domain within a single protein. See Supplementary spreadsheet 1 for details including definitions of domains, MIRs and NBRs. Source data are provided as a Source Data file.

a

b

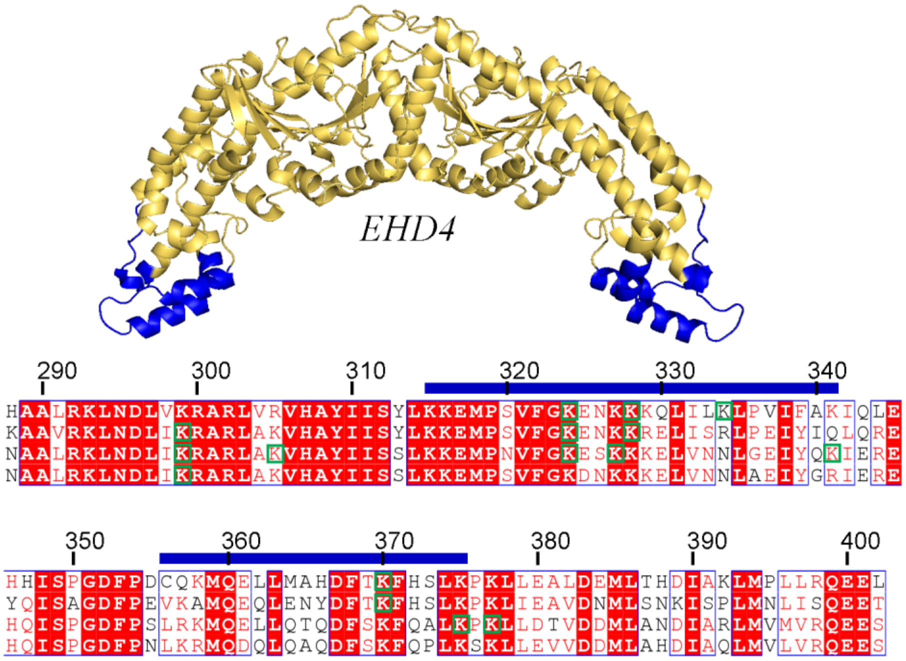

c

Gene name (.pdb)	MIR %K_{AC}	NBR %K_{AC}	Ratio
EHD2 (4CID)	8.51	0.00	MIR only
EHD4 (5MTV)	6.38	1.47	4.34
EHD1	8.51	4.41	1.93
EHD3	0.00	1.47	0.00
Average	5.85	1.84	3.18

Supplementary Figure 5. **Eps15-homology EHD1-4 domains localize acetylation to their membrane-interaction regions.** A) The crystal structure of EHD4 (5MTV.pdb) with MIRs and NBRs colored blue and gold, respectively. B) Sequence alignments of EHD 1, 3, and 4 against EHD2 (288-402). MIRs are demarcated by a blue bar and acetylated lysines are denoted by a green box. C) Prevalence of acetylation in MIRs and NBRs for EHD1-4. Percentages represent the number of acetylated lysine residues out of the total amino acid residues in their respective regions. (.pdb) file names indicate the PDB crystal structure used. See Supplementary spreadsheet 1 for details including definitions of domains, MIRs and NBRs. Source data are provided as a Source Data file.

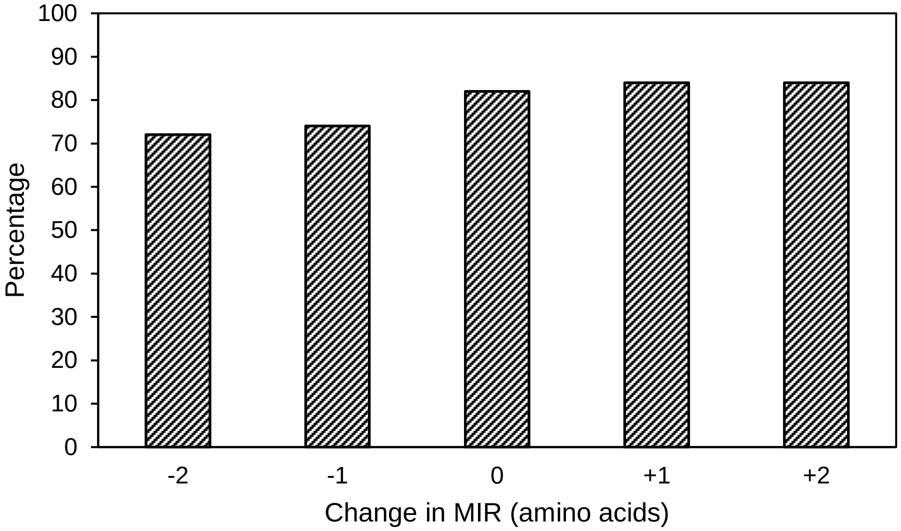

Supplementary Figure 6. **Predominance of lysine acetylation in the membrane-interaction regions remains largely unchanged upon expansion and contraction of MIR definitions.** Domain definitions were increased or decreased by 1 or 2 amino acids for each domain analyzed and the total percentage of domains within the data set for which lysine acetylation was predominant within the MIR is shown for each condition. Source data are provided as a Source Data file.

	Total K			Total K_{AC}			%K		
Domain	MIR	NBR	Ratio	MIR	NBR	Ratio	MIR	NBR	Ratio
**BAR**	**220**	**102**	**2.2**	**45**	**10**	**4.5**	**15.6**	**6.3**	**2.5**
**PX**	**76**	**54**	**1.4**	**20**	**5**	**4.0**	**10.7**	**6.2**	**1.7**
**C2**	**144**	**57**	**2.5**	**33**	**6**	**5.5**	**14.4**	**6.4**	**2.3**
**EHD**	**38**	**18**	**2.1**	**11**	**5**	**2.2**	**20.2**	**6.6**	**3.1**
**Total**	**478**	**231**	**2.1**	**109**	**26**	**4.2**	**14.5**	**6.3**	**2.3**

Supplementary Table 1. **Contributing factors to the prevalence of lysine acetylation by domain family in membrane-interaction regions and non-binding regions.** Details from the analysis are presented, including the total number of lysines (Total K) and the total number of acetylated lysines (Total K_{AC}) found within MIRs and NBRs of each domain family. K, number of lysine residues; K_{AC}, number of acetylated lysine residues; %K, number of lysine residues normalized by the total amino acid in the region; MIR, membrane-interaction region; NBR, non-binding region. See Supplementary spreadsheet 1 for details including definitions of domains, MIRs and NBRs. Source data are provided as a Source Data file.

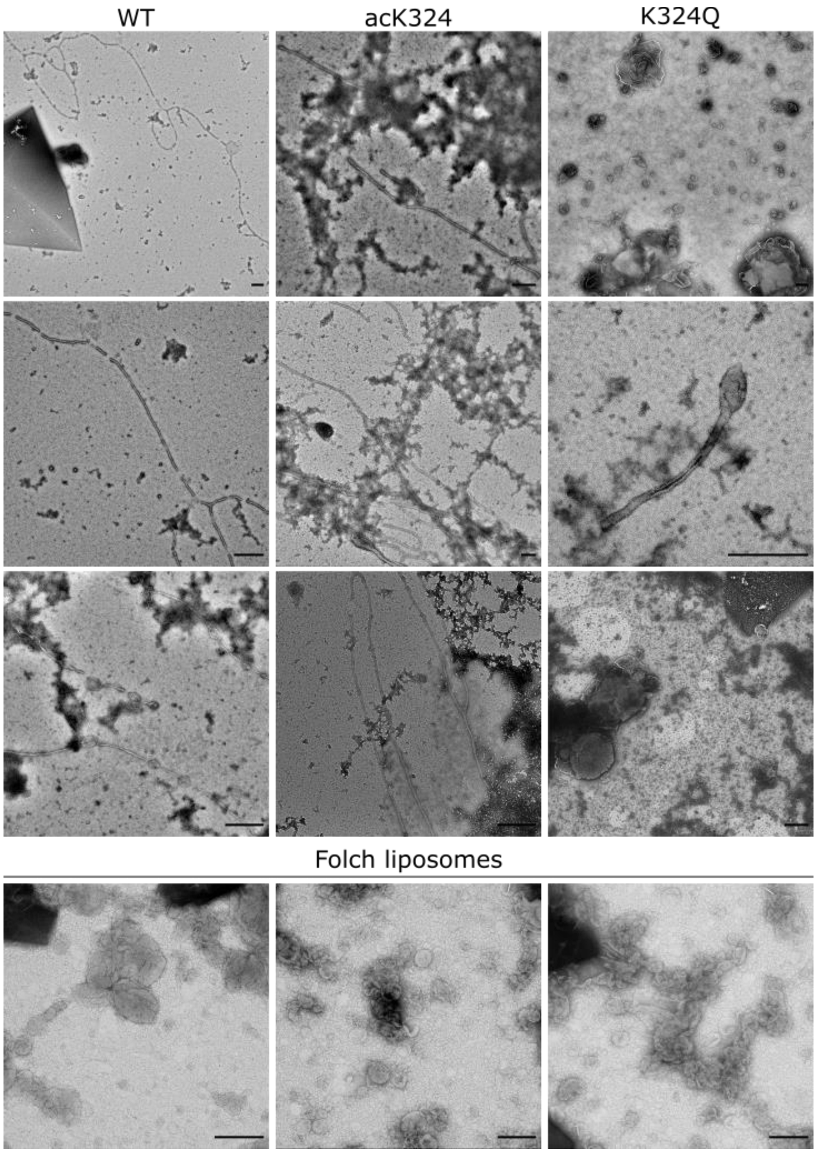

Supplementary Figure 7. **TEM micrographs showing EHD2-mediated membrane remodeling at a concentration of 10 µM.** Representative images are shown for WT, EHD2-acK324 and EHD2-KK324Q variants incubated with Folch liposomes. Long and intricate networks of lipid tubules decorated with protein were found for the WT and acetylated constructs. Intriguingly, EHD2-acK324 showed some detached membranous particles decorated with protein, which were not apparent for EHD2-WT. The remodeling activity for the glutamine mimic was greatly decreased and only one short tubule was found at this concentration. Scale bars = 500nm. Micrographs were repeated three times each in three independent assays.

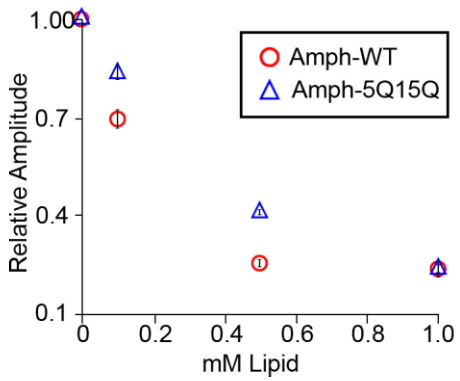

Supplementary Figure 8. **Mimicking Acetylation reducing membrane binding affinity of Amphiphysin.** Mean values of EPR spectral amplitudes are plotted as a function of lipid concentration for Amph-WT (solid line) and Amph-5Q15Q (dashed line). Error bars represent the range (s.e.m.) of 3 independent experiments. n = 3. Source data are provided as a Source Data file.

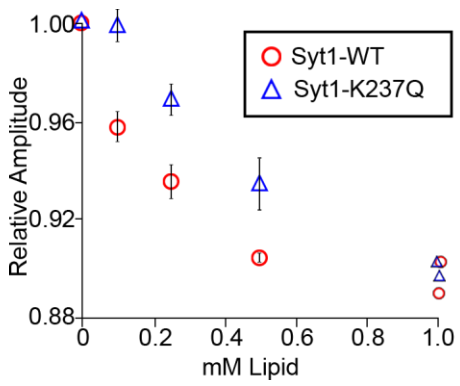

Supplementary Figure 9. **Mimicking acetylation reduces synaptotagmin1 membrane binding affinity.** Mean values of EPR spectral amplitudes are plotted as a function of lipid concentration for Syt1-WT (solid line) and Syt1-K237Q (dashed line). Error bars represent the range (s.e.m.) of 3 independent experiments, (i.e. n = 3). Where no error bar is shown at 1mM lipid, individual data points are shown instead. Source data are provided as a Source Data file.

Supplementary Table 2. **List of Primers.**

Primer Description	Primer Sequence	Notes
dAmphi_K5Q_fwd	CACCATATGACCGAAAATCAGGGCATAATGCTGGCC	used for the A20 (TEM) and A20C-R1 (lipid saturation curves). Built the 5Q, 15Q construct one mut at a time with 15Q primer
dAmphi_K5Q_rev	GGCCAGCATTATGCCCTGATTTTCGGTCATATGGTG	used for the A20 (TEM) and A20C-R1 (lipid saturation curves). Built the 5Q, 15Q construct one mut at a time with 15Q primer
dAmphi_K15Q_fwd	GCTGGCCAAATCTGTTCAACAGCACGCTGGACG	used for the A20 (TEM) and A20C-R1 (lipid saturation curves). Built

		the 5Q, 15Q construct one mut at a time with 5Q primer
dAmphi_K15Q_rev	CGTCCAGCGTGCTGTTGAACAGATTTGGCCAGC	used for the A20 (TEM) and A20C-R1 (lipid saturation curves). Built the 5Q, 15Q construct one mut at a time with 5Q primer
dAmphi_K5R_fwd	CACCATATGACCGAAAATCGTGGCATAATGCTGGCC	used for the A20 (TEM), Built the 5R, 15R construct one mut at a time with 15R primer
dAmphi_K5R_rev	GGCCAGCATTATGCCACGATTTTCGGTCATATGGTG	used for the A20 (TEM), Built the 5R, 15R construct one mut at a time with 15R primer
dAmphi_K15R_fwd	GCTGGCCAAATCTGTTCAACGGCACGCTGGACG	used for the A20 (TEM), Built the 5R, 15R construct one mut at a time with 5R primer
dAmphi_K15R_rev	CGTCCAGCGTGCCGTTGAACAGATTTGGCCAGC	used for the A20 (TEM), Built the 5R, 15R construct one mut at a time with 5R primer
Fwd- NheI Amphi pEGFP	CCTGCATCATCACCATCAGCTAGCCCATATGACCG	used to introduce cut sites for putting dAmphi BAR domain wt and amphi5Q_15Q into pEGFP vector for confocal studies
Rev- XhoI Amphi pEGFP	GGTGATGATGCTCGAGGGAGCCGCGCTGCGATTCTGTGGCC	used to introduce cut sites for putting dAmphi BAR domain wt and amphi5Q_15Q into pEGFP vector for confocal studies
Fwd- XhoI Amphi pINDY6	CCTGCATCATCACCATCACTCGAGCCATATGACCG	used to introduce cut sites for putting dAmphi BAR domain wt and amphi5Q_15Q into pINDY6 for drosophila studies (Karen's work)
Rev- SpeI Amphi pINDY6	GGTGATGATGACTAGTTTAGGAGCCGCGCTGCGATTCTGTG GCC	used to introduce cut sites for putting dAmphi BAR domain wt and amphi5Q_15Q into pINDY6 for drosophila studies (Karen's work)
Fwd- XhoI FlAmphi pINDY6	CCGCGTGGATCCCCAGGAATTCGACTCGAGATGACCG	used to introduce cut sites for putting full length dAmphi wt and amphi5Q_15Q into pINDY6 for drosophila studies (Karen's work)
Rev- SpeI FLamphi pINDY6	GGCCGCTGGCGACTAGTTCAGATGGGACGCG	used to introduce cut sites for putting full length dAmphi wt and amphi5Q_15Q into pINDY6 for drosophila studies (Karen's work)
Fwd-EHD2 K324Q	GCCCACGGTGTTCGGGCAGGAAAACAAGAAGAAGCAGCTG ATCC	used to introduce K324Q into wt EHD2 (TEM) and 321C-R1 (lipid saturation)
Rev-EHD2 K324Q	GGATCAGCTGCTTCTTCTTGTTTTCCTGCCCGAACACCGTGG GC	used to introduce K324Q into wt EHD2 (TEM) and 321C-R1 (lipid saturation)
Fwd-EHD2 K328Q	GCCCACGGTGTTCGGGAAGGAAAACAAGCAGAAGCAGCTG ATCC	used to introduce K328Q into wt EHD2 (TEM)
Rev-EHD2 K328Q	GGATCAGCTGCTTCTGCTTGTTTTCCTTCCCGAACACCGTGG GC	used to introduce K328Q into wt EHD2 (TEM)

Fwd-EHD2 K324Q K328Q	GCCCACGGTGTTCGGGCAGGAAAACAAGCAGAAGCAGCTGATCC	used to introduce K324Q K328Q into wt EHD2 (TEM) and construct for pEGFP confocal microscopy work
Rev-EHD2 K324Q K328Q	GGATCAGCTGCTTCTGCTTGTTTTCCTGCCCGAACACCGTGGGC	used to introduce K324Q K328Q into wt EHD2 (TEM) and construct for pEGFP confocal microscopy work
Fwd- Stg K237Q	GATCGCTTCTCCCAGCACGACATCATCGGAGAG	used to mutate K237Q on wt Syt1 for TEM and on 227C-R1 for lipid saturation curves
Rev- Stg K237Q	CTCTCCGATGATGTCGTGCTGGGAGAAGCGATC	used to mutate K237Q on wt Syt1 for TEM and on 227C-R1 for lipid saturation curves
Fwd-5Q15Q FLdAmphi	CCGAAAATCAGGGCATAATGCTGGCCAAATCTGTTCAACAGCACGCTGGACGTGCC	FL dAmphi
rev-5Q15Q FLdAmphi	GGCACGTCCAGCGTGCTGTTGAACAGATTTGGCCAGCATTATGCCCTGATTTTCGG	FL dAmphi
Gibson_Amph_forward	CTTTAATAAGGAGATATACCATGACCGAAAATAAAGGC	Gibson assembly into pRSF-Duet1/AcKRS3/pylT (vector also encoding for the synthetically evolved tRNA synthetase/tRNA-pair from Methanosarcina barkeri as described in the Material and Methods section.
Gibson_Amph_reverse	CGCAGCAGCGGTTTCTTTACTTAATGGTGATGGTGATG	Gibson assembly into pRSF-Duet1/AcKRS3/pylT (vector also encoding for the synthetically evolved tRNA synthetase/tRNA-pair from Methanosarcina barkeri as described in the Material and Methods section.
Gibson_EHD2_forward	CTTTAATAAGGAGATATACCATGGGCAGCAGCCATCATC	Gibson assembly into pRSF-Duet1/AcKRS3/pylT (vector also encoding for the synthetically evolved tRNA synthetase/tRNA-pair from Methanosarcina barkeri as described in the Material and Methods section.
Gibson_EHD2_reverse	CGCAGCAGCGGTTTCTTTACTTATTCAGCAGAGCCCTTCTG	Gibson assembly into pRSF-Duet1/AcKRS3/pylT (vector also encoding for the synthetically evolved tRNA synthetase/tRNA-pair from Methanosarcina barkeri as described in the Material and Methods section.
mmEHD2 K324Q	FWD:G ATG CCC ACG GTT TTC GGG cag GAA AAC AAG AAG AAG CAG	mmEHD2 K324Q.
mmEHD2 K324Q	REV:CTG CTT CTT CTT GTT TTC ctg CCC GAA AAC CGT GGG CAT C	mmEHD2 K324Q.
**EHD2 V321C Fwd**	AAAGAGATGCCCACGTGCTTCGGGTAGGAAAAC	**For Construction of EHD V321C**
**EHD2 V321C rev**	GTTTTCCTACCCGAAGCACGTGGGCATCTCTTT	**For Construction of EHD V321C**

**EHD2 K324 amber For**	GATGCCCACGGTGTTCGGGTAGGAAAACAAGAAGAAGCAGC	**EHD2 K324 amber**
**EHD2 K324 amber Rev**	GCTGCTTCTTCTTGTTTTCCTACCCGAACACCGTGGGCATC	**EHD2 K324 amber**
**EHD2 C96S For**	GAGCCCACCACCGACAGCTTCGTGGCTGTCATG	**EHD2 C96S**
**EHD2 C96S Rev**	CATGACAGCCACGAAGCTGTCGGTGGTGGGCTC	**EHD2 C96S**
**EHD2 C138S For**	CTCAACAGGTTTATGAGCGCCCAACTCCCCAAC	**EHD2 C138S**
**EHD2 C138S Rev**	GTTGGGGAGTTGGGCGCTCATAAACCTGTTGAG	**EHD2 C138S**
**EHD2 C356S For**	GGAGACTTTCCTGACAGCCAGAAGATGCAGGAG	**EHD2 C356S**
**EHD2 C356S Rev**	CTCCTGCATCTTCTGGCTGTCAGGAAAGTCTCC	**EHD2 C356S**
