# Supplementary material for: Lysine acetylation regulates the interaction between proteins and membranes
Source: Nat Commun. 2021 Nov 9;12:6466. doi: 10.1038/s41467-021-26657-2 (PMC8578602; doi:10.1038/s41467-021-26657-2)
Supplement: Supplementary file 3 — Description of Additional Supplementary Files [file 41467_2021_26657_MOESM3_ESM.pdf]

Description of Additional Supplementary Files

File name: Supplementary Data 1

Description: Code for bioinformatics structural and Matlab data analysis.
