# Supplementary material for: Lysine acetylation regulates the interaction between proteins and membranes
Source: Nat Commun. 2021 Nov 9;12:6466. doi: 10.1038/s41467-021-26657-2 (PMC8578602; doi:10.1038/s41467-021-26657-2)
Supplement: Supplementary file 5 — Reporting Summary [file 41467_2021_26657_MOESM5_ESM.pdf]

nature research

Corresponding author(s): Alan Okada

Last updated by author(s): May 21, 2021

Reporting Summary

Nature Research wishes to improve the reproducibility of the work that we publish. This form provides structure for consistency and transparency in reporting. For further information on Nature Research policies, see our Editorial Policies and the Editorial Policy Checklist.

Statistics

For all statistical analyses, confirm that the following items are present in the figure legend, table legend, main text, or Methods section.

n/a	Confirmed	
☐	☒	The exact sample size (*n*) for each experimental group/condition, given as a discrete number and unit of measurement
☐	☒	A statement on whether measurements were taken from distinct samples or whether the same sample was measured repeatedly
☒	☐	The statistical test(s) used AND whether they are one- or two-sided<br>*Only common tests should be described solely by name; describe more complex techniques in the Methods section.*
☒	☐	A description of all covariates tested
☒	☐	A description of any assumptions or corrections, such as tests of normality and adjustment for multiple comparisons
☐	☒	A full description of the statistical parameters including central tendency (e.g. means) or other basic estimates (e.g. regression coefficient) AND variation (e.g. standard deviation) or associated estimates of uncertainty (e.g. confidence intervals)
☒	☐	For null hypothesis testing, the test statistic (e.g. *F*, *t*, *r*) with confidence intervals, effect sizes, degrees of freedom and *P* value noted<br>*Give P values as exact values whenever suitable.*
☒	☐	For Bayesian analysis, information on the choice of priors and Markov chain Monte Carlo settings
☒	☐	For hierarchical and complex designs, identification of the appropriate level for tests and full reporting of outcomes
☒	☐	Estimates of effect sizes (e.g. Cohen's *d*, Pearson's *r*), indicating how they were calculated

Our web collection on statistics for biologists contains articles on many of the points above.

Software and code

Policy information about availability of computer code

Data collection	For data collection, lysine acetylation data were extracted from a database downloaded from phosphosite.org. These data were aligned with structural and sequence data for the relevant proteins using in-house code. This code reads the phosphosite.org database, matches the entries to structural (PDB ID) and sequence (FASTA) data via the "PDB cross-references in UniProtKB/Swiss-Prot" document at the SwissProt site. The in-house code is freely available on request.
Data analysis	For data analysis, the in-house code outputs the sequences with lysine acetylation and provides images (Pymol scripts) showing the location of the acetylation. This approach was used to initiate further analyses that were performed in Excel. The in-house code is freely available on request. All other related software is commonly available or can be substituted by other similar software.

For manuscripts utilizing custom algorithms or software that are central to the research but not yet described in published literature, software must be made available to editors and reviewers. We strongly encourage code deposition in a community repository (e.g. GitHub). See the Nature Research guidelines for submitting code & software for further information.

Data

Policy information about availability of data

All manuscripts must include a data availability statement. This statement should provide the following information, where applicable:

- Accession codes, unique identifiers, or web links for publicly available datasets
- A list of figures that have associated raw data
- A description of any restrictions on data availability

The authors confirm that the data supporting the findings of this study are available within the article [and/or] its supplementary materials.

Field-specific reporting

Please select the one below that is the best fit for your research. If you are not sure, read the appropriate sections before making your selection.

☒ Life sciences ☐ Behavioural & social sciences ☐ Ecological, evolutionary & environmental sciences

For a reference copy of the document with all sections, see nature.com/documents/nr-reporting-summary-flat.pdf

Life sciences study design

All studies must disclose on these points even when the disclosure is negative.

Sample size	Sample size is shown in the figure, figure legends, or in the methods section. No statistical method was used to pre-determine sample size, but our sample size was similar to previous studies.
Data exclusions	No data was excluded from the analysis.
Replication	All data are from a minimum of 5 independent experiments for drosophila studies. For all cell culture studies, data are from a minimum of 3 independent experiments.
Randomization	Samples were randomized during data collection.
Blinding	For drosophila studies, samples were randomized and data analyses were performed with the scorer blinded to the conditions and genotypes. Experiments were not performed with the researcher blinded to the conditions of the experiments or genotypes of the flies. For cell culture studies, the samples were randomized and researchers were blinded to the conditions during data collection.

Reporting for specific materials, systems and methods

We require information from authors about some types of materials, experimental systems and methods used in many studies. Here, indicate whether each material, system or method listed is relevant to your study. If you are not sure if a list item applies to your research, read the appropriate section before selecting a response.

Materials & experimental systems

n/a	Involved in the study	
☐	☒	Antibodies
☐	☒	Eukaryotic cell lines
☒	☐	Palaeontology and archaeology
☐	☒	Animals and other organisms
☒	☐	Human research participants
☒	☐	Clinical data
☒	☐	Dual use research of concern

Methods

n/a	Involved in the study	
☒	☐	ChIP-seq
☒	☐	Flow cytometry
☒	☐	MRI-based neuroimaging

Antibodies

Antibodies used	This information is described in the Methods section.
Validation	This information is described in the Methods section.

Eukaryotic cell lines

Policy information about cell lines

Cell line source(s)	HeLa and COS-7 cell lines were purchased from ATCC
Authentication	Authentication was performed by ATCC prior to purchase
Mycoplasma contamination	Mycoplamsa was not tested
Commonly misidentified lines (See ICLAC register)	None

Animals and other organisms

Policy information about studies involving animals; ARRIVE guidelines recommended for reporting animal research

Laboratory animals	Drosophila melanogaster was used in this study. Information on strains and generation of the fly lines used in this study are described in the Methods section.
Wild animals	The study did not involve wild animals
Field-collected samples	The study did not involve samples collected from the field
Ethics oversight	N/A

Note that full information on the approval of the study protocol must also be provided in the manuscript.
