# Supplementary material for: Lysine acetylation regulates the interaction between proteins and membranes
Source: Nat Commun. 2021 Nov 9;12:6466. doi: 10.1038/s41467-021-26657-2 (PMC8578602; doi:10.1038/s41467-021-26657-2)
Supplement: Supplementary file 4 — Supplementary Data 1 [file 41467_2021_26657_MOESM4_ESM.zip › Matlab.zip!/K_Kac_Alanalysis.m]

%% S1 structure with region vectors and corresponding protein names. 
clc;clear;load([date,'-ver8']); 
xf=fieldnames(data2);
dataRegionScalar=rmfield(data2,xf(1:9));	
xf=fieldnames(dataRegionScalar);		
c=1;
for i=2:2:length(xf)
    xa=xf{i};
    xb=xf{i+1};
    z{c}={xa,xb};%obtain field name
    c=c+1;
end
f={'D','M1','M2','M3'};
for p=1:cf
    for i=1:4
        xa=z{i};
        xb=dataRegionScalar(p).(xa{1});
        xc=dataRegionScalar(p).(xa{2});
        S1(p).(f{i})=[xb,xc]; %start end vector
    end
end
%adding protein names to the region vector
xf=fieldnames(dataRegionScalar);
S2=rmfield(dataRegionScalar,xf(2:length(xf)));
S2(52)=[];S2(52)=[];%artifact empty entry on the structure is removed
S1=fieldtransfer(S2,'protein',S1,'protein');%add protein name to the region vector
save([date,'-ver9']); 
%% adding NMBR
clc;clear;load([date,'-ver9']); 
for i=1:cf
     if isempty(S1(i).M3)==0
         S1(i).N0=[S1(i).D(1),S1(i).M1(1)-1];
         S1(i).N1=[1+S1(i).M1(2),S1(i).M2(1)-1];
         S1(i).N2=[1+S1(i).M2(2),S1(i).M3(1)-1];
         S1(i).N3=[1+S1(i).M3(2),S1(i).D(2)];
     elseif isempty(S1(i).M2)==0
         S1(i).N0=[S1(i).D(1),S1(i).M1(1)-1];
         S1(i).N1=[1+S1(i).M1(2),S1(i).M2(1)-1];
         S1(i).N2=[1+S1(i).M2(2),S1(i).D(2)];
     elseif isempty(S1(i).M1)==0
         S1(i).N0=[S1(i).D(1),S1(i).M1(1)-1];
         S1(i).N1=[1+S1(i).M1(2),S1(i).D(2)];
     end
end
save([date,'-ver10']); 
%% remove NMBR with length <=1 due to collision with the domain; see DYSF i=38 N0
clc;clear;load([date,'-ver10']); % fist [1,1] into [];
f={'N0','N1','N2','N3'};
for j=1:4
for i=1:cf
    S2(i).(f{j})=rlength(S1(i).(f{j}));
    if (S2(i).(f{j}))<=1 % if the rigion vector has a length less than 1, which doenst make sense 
        S1(i).(f{j})=[]; %get rid of it 
        S2(i).(f{j})=[];%get rid of it 
    end  
end
end
save([date,'-ver11']); 
%% TR calculations
clc;clear;load([date,'-ver11']); 

tr=[0:4];
for i=1:length(tr)
    data4(i).transitionLength=tr(i);
    data4(i).vector=acetylationAnalysis3(S1,S2,cf,tr(i));
end

save('keepGoing1.mat');%run multiple Tr values


%%
clc;clear; load('keepGoing1.mat');%

for t=1:length(tr)
    x=data4(t).vector;
    data5=x;
    xf=fieldnames(x);
    for i=1:cf
        seq=data(i).seq;
        for j=3:length(xf)
             v=x(i).(xf{j});
             if isempty(v)==0
                 data5(i).(xf{j})=getrl(seq,v(1),v(2));
             end
        end
    end
    data4(t).region=data5;   
end

save('keepGoing2.mat');%generate region (sequence from the vector)


%%
clc; clear; load('keepGoing2.mat');%
for i=1:cf
    x=data(i);
    seq=x.seq;
    allk=strfind(seq,'K');
    S3(i).allk=allk;
        S3(i).allk_count=length(allk);
    S3(i).kac_pos=[data(i).phos(1).Kac.pos];
            S3(i).kac_count=length([data(i).phos(1).Kac.pos]);
end

     save('keepGoing3.mat');% add All Lysines and Acetylated Lysines to data3% %%
%%
    clc;clear;load('keepGoing3.mat');
    for t=1:length(tr)
        data5=data4(t).vector;
                data5b=data4(t).vector;
        for i=1:cf
            xv=data4(t).vector;
            xk=S3(i).kac_pos;
            for j=3:length(xf)
                xb=xv(i).(xf{j});
                if isempty(xb)==0
                    xop=xk(xk>=xb(1) & xk<=xb(2));
                else
                    xop=[];
                end
                data5(i).(xf{j})=xop; 
                data5b(i).(xf{j})=length(xop);
            end
        end
        data4(t).kac_pos=data5;
                data4(t).kac_count=data5b;
    end
     save('keepGoing4.mat');% kac kacTotal analysis
     %%
    clc;clear;load('keepGoing4.mat');
    for t=1:length(tr)
        data5=data4(t).vector;
                data5b=data4(t).vector;
        for i=1:cf
            xv=data4(t).vector;
            xk=S3(i).allk;
            for j=3:length(xf)
                xb=xv(i).(xf{j});
                if isempty(xb)==0
                    xop=xk(xk>=xb(1) & xk<=xb(2));
                else
                    xop=[];
                end
                data5(i).(xf{j})=xop; 
                data5b(i).(xf{j})=length(xop);
            end
        end
        data4(t).allk_pos=data5;
                data4(t).allk_count=data5b;
    end
     save('keepGoing5.mat');% kac kacTotal analysis
     
    
     
     %%
     

clc;clear; load('keepGoing5.mat');%
xf4=fieldnames(data4);
data4(length(tr)+1).transitionLength='combined';

for i=2:7
xf=fieldnames(data4(1).(xf4{i}));
data5=struct([]);
for j=1:length(tr)
    for k=1:length(xf)
        data5=fieldtransfer(data4(j).(xf4{i}),xf{k},data5,[xf{k},'_',num2str(j)]);
    end
end
data4(length(tr)+1).(xf4{i})=data5;
end

save('keepGoing6.mat');%combined for export 
  
%%
clc;load('keepGoing6.mat');%combined for export 
clear('x');
xf2=fieldnames(data4);
for i=1:cf
    for j=3:length(xf2)
        for t=1:length(tr)
            data6(i).(xf2{j})(t)=data4(t).(xf2{j})(i);
        end
    end
end
